# Development of Chronic Thyroiditis During Cyclosporin A Treatment

**DOI:** 10.1210/jcemcr/luae211

**Published:** 2024-11-20

**Authors:** Yuji Hataya, Takumi Nomura, Yuko Fujishima, Kanta Fujimoto, Toshio Iwakura, Naoki Matsuoka

**Affiliations:** Department of Diabetes and Endocrinology, Kobe City Medical Center General Hospital 2-1-1, Kobe, Hyogo 650-0047, Japan; Department of Diabetes and Endocrinology, Kobe City Medical Center General Hospital 2-1-1, Kobe, Hyogo 650-0047, Japan; Department of Diabetes and Endocrinology, Kobe City Medical Center General Hospital 2-1-1, Kobe, Hyogo 650-0047, Japan; Department of Diabetes and Endocrinology, Kobe City Medical Center General Hospital 2-1-1, Kobe, Hyogo 650-0047, Japan; Department of Diabetes and Endocrinology, Kobe City Medical Center General Hospital 2-1-1, Kobe, Hyogo 650-0047, Japan; Department of Diabetes and Endocrinology, Kobe City Medical Center General Hospital 2-1-1, Kobe, Hyogo 650-0047, Japan

**Keywords:** chronic thyroiditis, MALT lymphoma, cyclosporin A

## Abstract

Cyclosporin A (CsA) is a calcineurin inhibitor used as an immunosuppressant. Although CsA effectively suppresses T cells, excessive suppression of regulatory T cells may exacerbate autoimmune diseases. Here, we report a case of chronic thyroiditis developing during CsA treatment. A 64-year-old woman, on CsA for 2 years for aplastic anemia, presented with a nodule in the right thyroid lobe, raising concern for malignant lymphoma. Right hemithyroidectomy confirmed mucosa-associated lymphoid tissue lymphoma without chronic thyroiditis in the adjacent normal tissue. Owing to the localized lesion, the patient was monitored with a reduced dose of CsA. Initial thyroid ultrasonography showed a normal left lobe; however, hypoechoic areas appeared 1-year postsurgery, followed by diffuse thyroid enlargement and further expansion of these hypoechoic areas. Postoperative fluorodeoxyglucose positron emission tomography showed progressive uptake in the left lobe, and thyroid autoantibodies, initially negative, became positive. Five years later, suspected lymphoma recurrence prompted a residual thyroidectomy, which confirmed mucosa-associated lymphoid tissue lymphoma with chronic thyroiditis. This case suggests that excessive suppression of regulatory T cells by CsA may induce chronic thyroiditis. Further studies on chronic thyroiditis in patients treated with CsA may enhance our understanding of its pathogenesis.

## Introduction

Cyclosporin A (CsA) is a calcineurin inhibitor and an immunosuppressant that inhibits T-cell differentiation, proliferation, and cytokine production [1]. CsA is widely used to prevent transplant rejection and graft-versus-host disease. It is also used to treat autoimmune disorders such as atopic dermatitis, nephrotic syndrome, and rheumatoid arthritis [1]. Furthermore, CsA is the first-line treatment for older adults with aplastic anemia and those lacking a suitable sibling donor [2]. Although CsA effectively suppresses T cells, it has been suggested that excessive suppression of regulatory T cells (Tregs) may exacerbate autoimmune diseases [3]. There are case reports of CsA-associated thyroid disorders, including Graves disease [4-9], thyroid-eye disease [10], and subacute thyroiditis [11]. However, no reports exist on the association between CsA and chronic thyroiditis.

Here, we report a case of a patient who developed chronic thyroiditis and thyroid mucosa-associated lymphoid tissue (MALT) lymphoma during CsA treatment. This case provides valuable insight into the mechanisms underlying the development of chronic thyroiditis.

## Case Presentation

A 64-year-old woman was referred to our department following the incidental discovery of a thyroid nodule during carotid artery ultrasonography. She had been diagnosed with aplastic anemia 2 years earlier and had been stable on CsA (200 mg/day). The patient had no family history of thyroid disease or autoimmune disorders and used no concomitant medications other than CsA.

## Diagnostic Assessment

**Figure 1.**
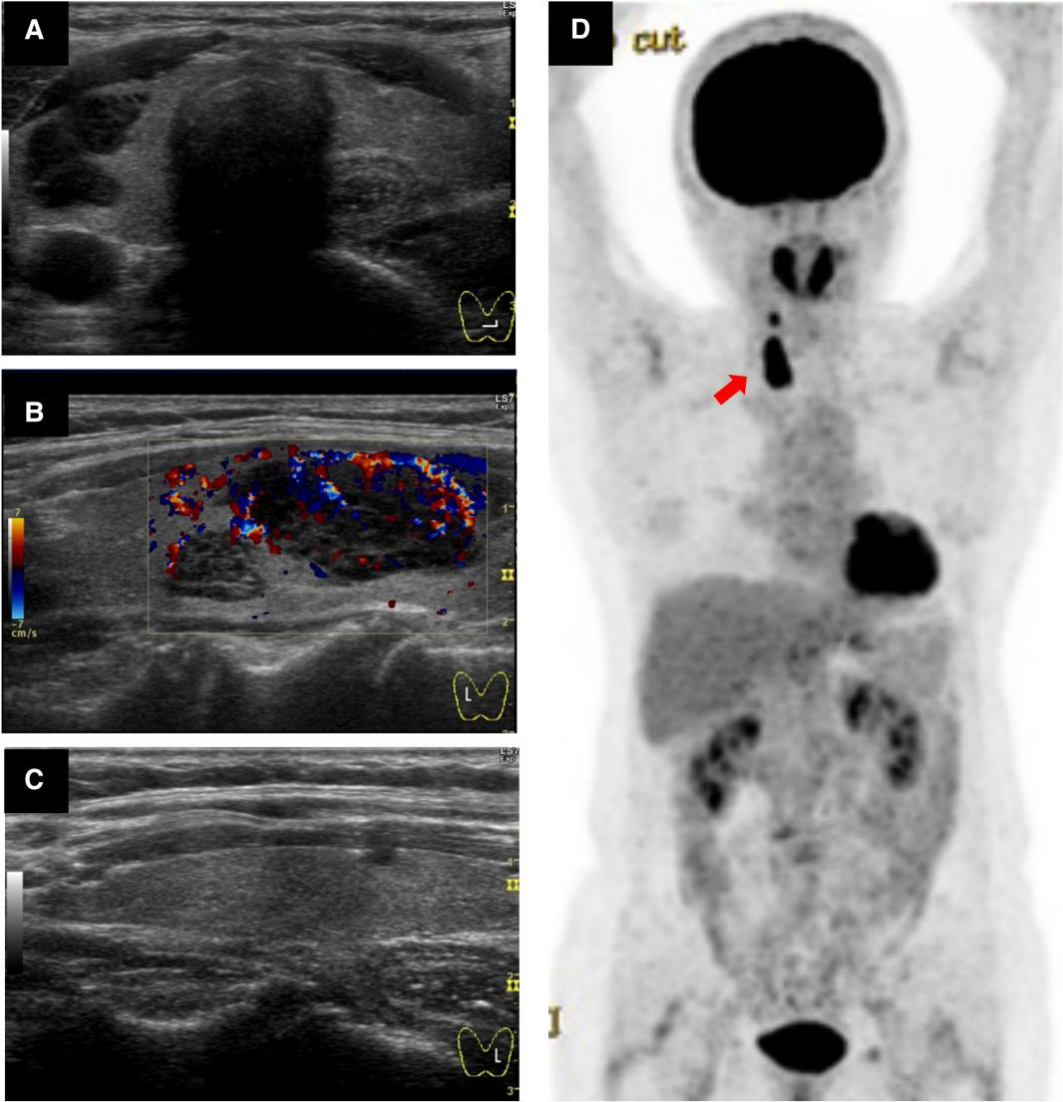
Imaging at the first surgery. (A-C) Ultrasound examination showed hypoechoic masses in the right thyroid lobe, measuring 25 mm × 19 mm and 11 mm × 9 mm. The border was well-defined, with the margins exhibiting a broccoli-like growth pattern. The left lobe appeared normal. (D) Fluorodeoxyglucose positron emission tomography revealed intense uptake corresponding to the masses in the right thyroid lobe (SUVmax 14.0), with no abnormal uptake elsewhere.

No significant masses or lymphadenopathy were detected in the neck. Thyroid ultrasound revealed hypoechoic masses in the right lobe, measuring 25 mm × 19 mm and 11 mm × 9 mm, suggestive of thyroid lymphoma (Fig. 1). The size and internal echogenicity of the left lobe were within normal limits. Blood tests revealed normal thyroid function and autoantibody levels (Table 1), with a soluble IL-2 receptor level of 244 U/mL (normal range, 220-530 U/mL). Her white blood cell count was 2.4 × 10^3^/μL (2.4 × 10^9^/L) (normal range, 3.9-9.8 × 10^3^/μL; 3.9-9.8 × 10^9^/L), red blood cell count was 327 × 10^4^/μL (327 × 10^10^/L) (normal range, 350-510 × 10^4^/μL; 350-510 × 10^10^/L), and platelet count was 10.8 × 10^4^/μL (10.8 × 10^10^/L) (normal range, 13.0-37.0 × 10^4^/μL; 13.0-37.0 × 10^10^/L). Fine-needle aspiration cytology identified numerous lymphocytes; however, it did not provide a definitive diagnosis. A suspected thyroid lymphoma diagnosis prompted fluorodeoxyglucose positron emission tomography (FDG-PET), which revealed intense uptake in the thyroid mass in the right lobe with no other abnormal uptake (Fig. 1).

**Table 1.** Blood examination summary over time

	At the first surgery	After the first surgery					Normal range
		1 y	2 y	3 y	4 y	5 y	
TSH	1.26 μIU/mL	2.46 μIU/mL	2.74 μIU/mL	2.84 μIU/mL	3.18 μIU/mL	2.85 μIU/mL	0.61-4.23 μIU/mL
	(1.26 mIU/L)	(2.46 mIU/L)	(2.74 mIU/L)	(2.84 mIU/L)	(3.18 mIU/L)	(2.85 mIU/L)	(0.61-4.23 mIU/L)
FT4	1.27 ng/dL	1.08 ng/dL	1.23 ng/dL	1.04 ng/dL	1.10 ng/dL	0.97 ng/dL	0.9-1.7 ng/dL
	(16.3 pmol/L)	(13.9 pmol/L)	(15.8 pmol/L)	(13.4 pmol/L)	(14.2 pmol/L)	(12.5 pmol/L)	(11.6-21.9 pmol/L)
TgAb	28 IU/mL	22 IU/mL	99 IU/mL	64 IU/mL	54 IU/mL	205 IU/mL	<28 IU/mL
	(28 IU/mL)	(22 IU/mL)	(99 IU/mL)	(64 IU/mL)	(54 IU/mL)	(205 IU/mL)	(<28 IU/mL)
TPOAb	11 IU/mL	10 IU/mL	11 IU/mL	NA	NA	<9 IU/mL	<16 IU/mL
	(11 IU/mL)	(10 IU/mL)	(11 IU/mL)			(<9 IU/mL)	(<16 IU/mL)
Tg	3.7 ng/mL	2.3 ng/mL	1.3 ng/mL	2.0 ng/mL	3.0 ng/mL	6.4 ng/mL	0.0-33.7 ng/mL
	(3.7 μg/L)	(2.3 μg/L)	(1.3 μg/L)	(2.0 μg/L)	(3.0 μg/L)	(6.4 μg/L)	(0.0-33.7 μg/L)
sIL-2R	244 U/mL	220 U/mL	248 U/mL	185 U/mL	252 U/mL	315 U/mL	220-530 U/mL
	(244 U/mL)	(220 U/mL)	(248 U/mL)	(185 U/mL)	(252 U/mL)	(315 U/mL)	(220-530 U/mL)

Values in parentheses are International System of Units (SI).

Abbreviations: FT4, free thyroxine; NA, not available; sIL-2R, soluble IL-2 receptor; Tg, thyroglobulin; TgAb, antithyroglobulin antibody; TPOAb, thyroid peroxidase antibody.

## Treatment

A right hemithyroidectomy was conducted for both diagnostic and therapeutic purposes.

## Outcome and Follow-up

**Figure 2.**
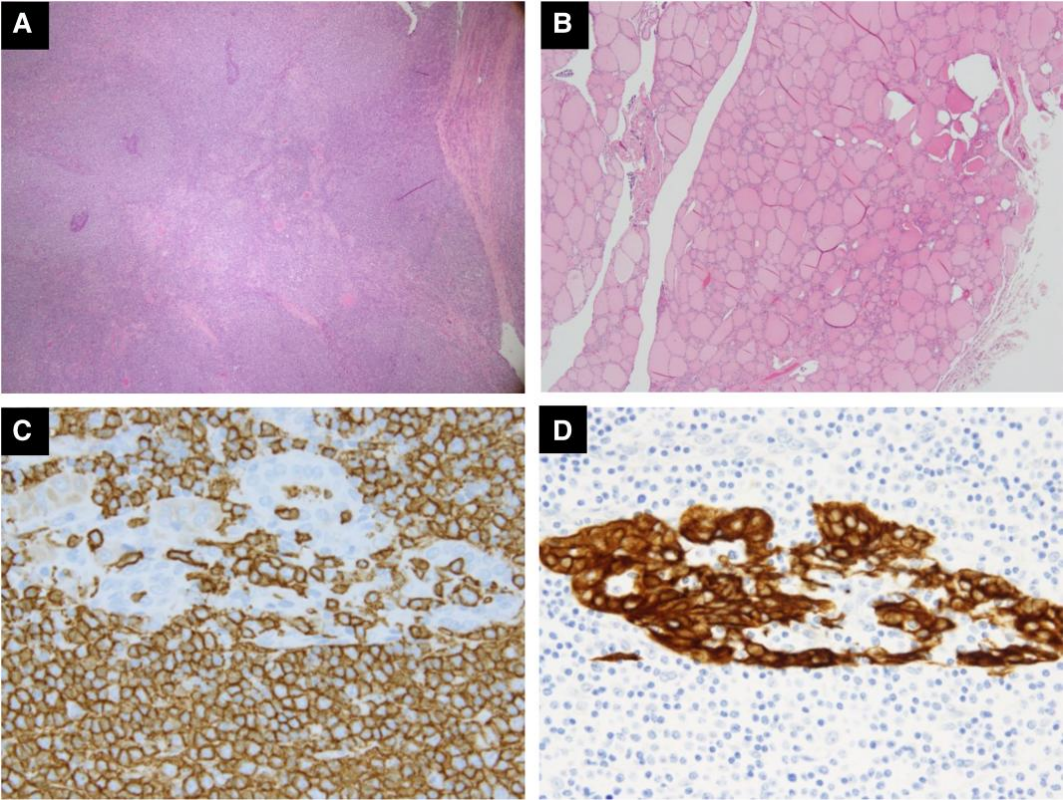
Histological and immunohistochemical findings of the right lobe specimen. (A) Dense infiltration of small- to medium-sized lymphocytes, as shown by hematoxylin and eosin staining (H&E). (B) No significant lymphocytic infiltration in the normal area with H&E staining. (C) Proliferation of CD20-positive B cells. (D) Lymphoepithelial lesions identified by cytokeratin staining.

Pathological examination revealed MALT lymphoma in the affected area, with no signs of chronic thyroiditis in the adjacent normal tissue (Fig. 2). Preoperative FDG-PET showed the lesion was confined to the right lobe. Consequently, no additional treatment was administered, and the patient was placed under observation. Considering the potential adverse effects of CsA, the dose was reduced to 150 mg. Subsequently, the patient's blood cell counts remained stable.

**Figure 3.**
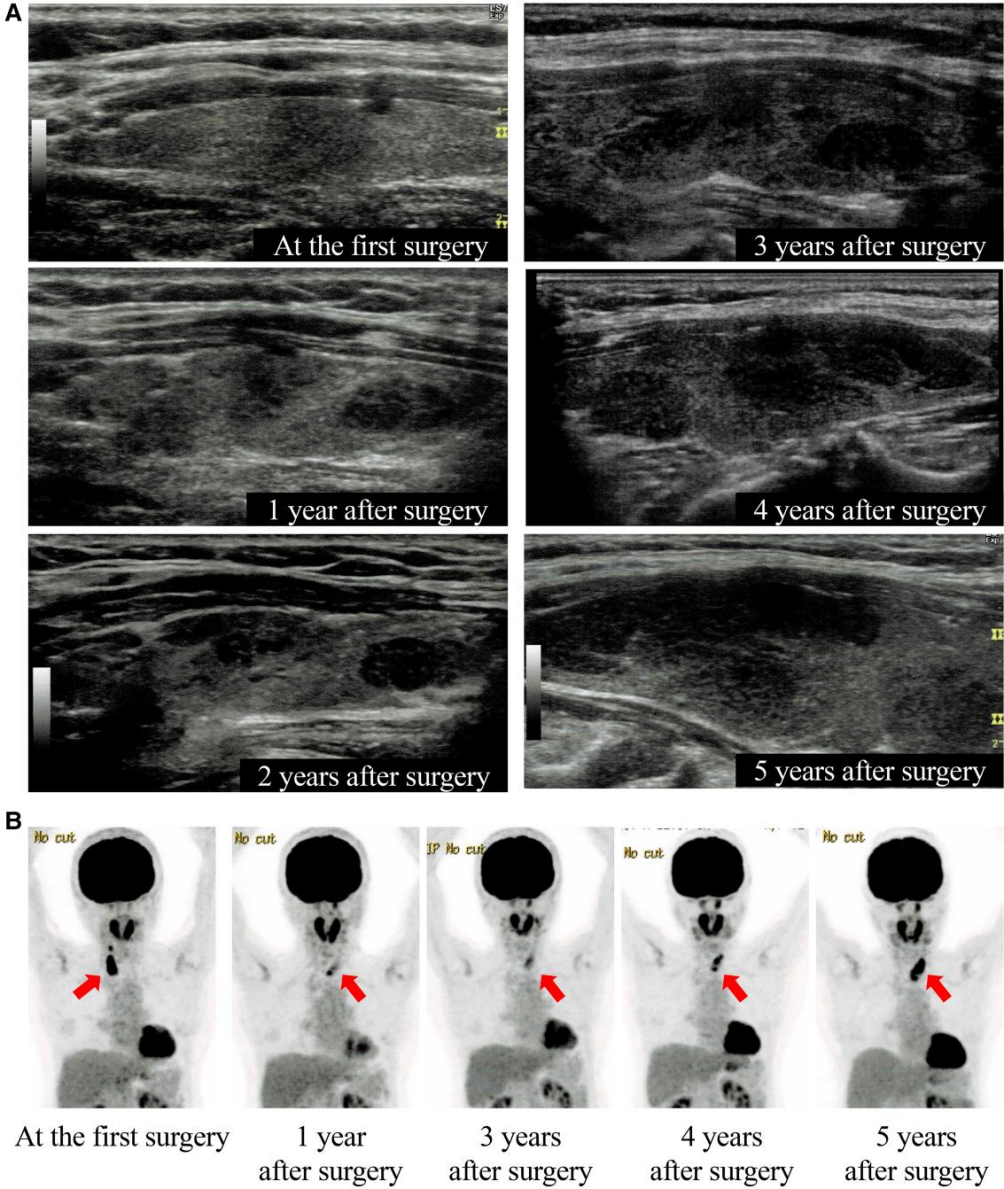
Changes in ultrasound and fluorodeoxyglucose positron emission tomography (FDG-PET) scans following the first surgery. (A) Ultrasound examination showed a hypoechoic area, which appeared in the left lobe 1 year postoperatively. Subsequently, this area enlarged, accompanied by heterogeneous diffuse goiter progression. (B) FDG-PET scan showed a progressive increase in diffuse uptake in the residual left lobe.

**Figure 4.**
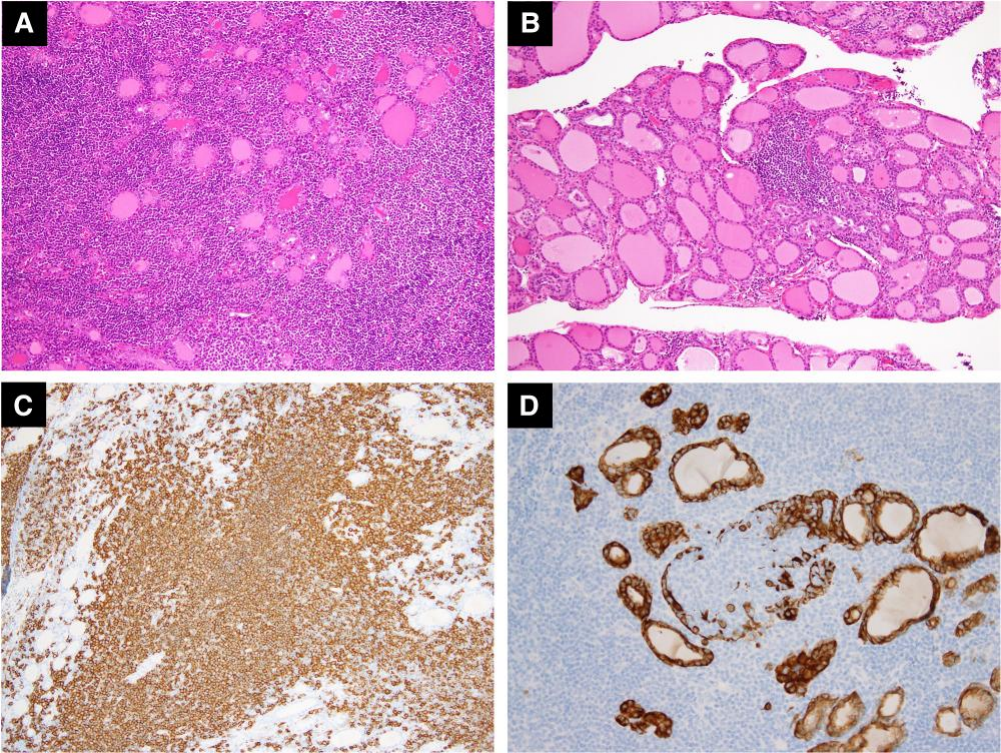
Histological and immunohistochemical findings of the left lobe specimen. (A) Dense infiltration of small- to medium-sized lymphocytes with lymphoid follicles, as demonstrated by hematoxylin and eosin (H&E) staining. (B) Surrounding thyroid tissue showed chronic thyroiditis, confirmed by H&E staining. (C) Proliferation of CD20-positive B cells. (D) Lymphoepithelial lesions identified by cytokeratin staining.

One year later, ultrasonography revealed a hypoechoic area in the left lobe, which expanded further alongside diffuse thyroid enlargement. Annual FDG-PET scans demonstrated progressive diffuse uptake, consistent with the ultrasound findings (Fig. 3). Over time, blood tests revealed increased thyroglobulin antibodies, whereas thyroid function remained stable (Table 1). The soluble IL-2 receptor levels, which were measured to monitor lymphoma recurrence, remained within the normal range. Initially, the patient was diagnosed with chronic thyroiditis and observed. However, 5 years later, fine-needle aspiration cytology suggested malignant lymphoma, leading to residual thyroidectomy. Pathological examination confirmed MALT lymphoma with surrounding chronic thyroiditis (Fig. 4). The patient continued CsA treatment, and no lymphoma recurrence has been observed.

## Discussion

In this case, the patient developed thyroid MALT lymphoma during CsA treatment for aplastic anemia, with no initial signs of chronic thyroiditis. However, continued CsA treatment worsened chronic thyroiditis and led to lymphoma recurrence, suggesting the role of CsA in these developments. To our knowledge, there are no previous reports of malignant lymphoma with chronic thyroiditis in patients receiving CsA treatment.

CsA binds to the cytoplasmic protein, cyclophilin, in T cells and inhibits calcineurin phosphatase activity. This inhibition prevents the activation of the nuclear factor of activated T cells [1], suppressing the development, activation, and differentiation of effector T cells, thereby leading to immunosuppressive effects. In addition, nuclear factor of activated T cells regulates FoxP3 expression and T-cell differentiation into Tregs [12]. Tregs play a crucial role in modulating effector T cells, maintaining immune tolerance to self-antigens, and suppressing inflammatory responses. Although CsA is generally effective as an immunosuppressant, it has been suggested that excessive suppression of Tregs may exacerbate autoimmune diseases [3].

Several case reports have documented an association between thyroid disorders and CsA treatment [4-11]. After hematopoietic stem cell transplantation, 10% to 50% of patients develop subclinical or overt hypothyroidism; this is attributed not only to pretransplant radiation therapy and graft-versus-host disease but also to the immunosuppressants administered during the transplantation process [13]. However, definitive data on chronic thyroiditis prevalence among patients receiving CsA treatment are lacking. Chronic thyroiditis is thought to result from genetic, environmental, and existential factors [14]. Recent studies have emphasized that an imbalance between T helper 17 (Th17) cells and Tregs plays a crucial role in autoimmune disease onset and progression [15]. In particular, Tregs dysfunction [16] and an imbalance between Th17 and Tregs [17, 18] have been linked to chronic thyroiditis. Therefore, excessive suppression of Tregs induced by CsA may have contributed to the development of chronic thyroiditis in this case. Further studies on patients who develop chronic thyroiditis during CsA treatment could help us better understand the pathogenesis of this condition.

CsA has long been associated with carcinogenesis owing to its immunosuppressive effects [19]. In this patient, we cannot exclude the role of CsA in the development of lymphoma. According to the 2017 World Health Organization classification, lymphomas developing in patients treated with immunosuppressants for autoimmune diseases are categorized as other iatrogenic immunodeficiency-associated lymphoproliferative disorders (OIIA-LPD) [20]. Although autoimmune diseases have a high frequency of LPD, the remission observed after immunosuppressant cessation suggests that these drugs play a role in OIIA-LPD development. Although most reported OIIA-LPD cases are associated with methotrexate (MTX) treatment for rheumatoid arthritis (MTX-LPD) [20], there are cases linked to CsA treatment (CsA-LPD) [21]. Although there are documented instances of MTX-LPD in the thyroid, no cases of CsA-LPD in the thyroid have been reported [22]. The mechanisms underlying OIIA-LPD include suppression of tumor immunity, activation of B cells resulting from Epstein-Barr virus (EBV) infection, and IL-6 activation [23]. In this patient, Epstein-Barr encoding region in situ hybridization for EBV was negative, indicating no EBV involvement. In addition, thyroid MALT lymphoma is frequently associated with chronic thyroiditis [24]; however, our patient showed no initial signs of chronic thyroiditis. Consequently, it remains unclear whether the lymphoma was due to chronic thyroiditis or CsA.

In conclusion, we report a case of chronic thyroiditis and thyroid MALT lymphoma that developed during CsA treatment for aplastic anemia. CsA exerts its immunosuppressive effects by inhibiting T-cell activation; however, it may induce autoimmune diseases by excessively suppressing Tregs. In this case, CsA may have contributed to chronic thyroiditis development. Further studies on CsA-induced chronic thyroiditis are needed for a better understanding of its pathogenesis.

## Learning Points

- CsA is an immunosuppressant commonly used to prevent transplant rejection and treat autoimmune disorders; however, it may exacerbate autoimmune diseases by excessively suppressing Tregs.
- An imbalance between Th17 and Tregs has been implicated in chronic thyroiditis, and the excessive suppression of Tregs induced by CsA may contribute to the development of this condition.
- CsA may potentially cause thyroid lymphoma as part of OIIA-LPD.

## Data Availability

Original data generated and analyzed during this study are included in this published article.

## Abbreviations

CsA: cyclosporin A
EBV: Epstein-Barr virus
FDG-PET: fluorodeoxyglucose positron emission tomography
MALT: mucosa-associated lymphoid tissue
MTX: methotrexate
OIIA-LPD: iatrogenic immunodeficiency-associated lymphoproliferative disorder
Th17: T helper 17
Treg: regulatory T cell
